# Crystal structure of Boc-(*S*)-ABOC-(*S*)-Ala-(*S*)-ABOC-(*S*)-Phe-OBn chloro­form monosolvate

**DOI:** 10.1107/S2056989015016941

**Published:** 2015-09-17

**Authors:** Emmanuel Wenger, Laure Moulat, Baptiste Legrand, Muriel Amblard, Monique Calmès, Claude Didierjean

**Affiliations:** a Université de Lorraine, UMR 7036 CRM2, Vandoeuvre-lès-Nancy, France; b CNRS, UMR 7036 CRM2, Vandoeuvre-lès-Nancy, France; c IBMM, UMR 5247 CNRS-Université Montpellier–ENSCM, 15 avenue Charles Flahault, 34093 Montpellier Cedex 5, France

**Keywords:** crystal structure, α,β-hybrid peptide, (*S*)-1-amino­bicyclo­[2.2.2]octane-2-carb­oxy­lic acid, (*S*)-ABOC, (*S*)-Ala, (*S*)-Phe, Boc, OBn, 11/9 helix, hydrogen bonding

## Abstract

In the title compound, phenyl (*S*)-2-[(*S*)-(1-{2-[(*S*)-(1-{[(*tert*-but­oxy)carbon­yl]amino}­bicyclo­[2.2.2]octan-2-yl)formamido]­propanamido}­bicyclo­[2.2.2]octan-2-yl)formamido]-3-phenyl­propano­ate chloro­form monosolvate, C_42_H_56_N_4_O_7_·CHCl_3_, the α,β-hybrid peptide contains two non-proteinogenic amino acid residues of (*S*)-1-amino­bicyclo­[2.2.2]octane-2-carb­oxy­lic acid [(*S*)-ABOC], two amino acid residues of (*S*)-2-amino­propanoic acid [(*S*)-Ala] and (*S*)-2-amino-3-phenyl­propanoic acid [(*S*)-Phe], and protecting groups of *tert*-but­oxy­carbonyl (Boc) and benzyl ester (OBn). The tetra­mer folds into a right-handed mixed 11/9 helix stabilized by intra­molecular *i*,*i* + 3 and *i*,*i* − 1 C=O⋯H—N hydrogen bonds. In the crystal, the oligomers are linked by N—H⋯O=C hydrogen bonds into chains along the *a-*axis direction. The chloro­form solvent mol­ecules are inter­calated between the folded chains *via* C—H⋯O=C inter­actions.

## Abstract 2

In the title compound, the α,β-hybrid peptide contains two non-proteinogenic amino acid residues [(*S*)-ABOC], two amino acid residues [(*S*)-Ala and (*S*)-Phe], and protecting groups of Boc and OBn. The tetra­mer folds into a right-handed mixed 11/9 helix stabilized by intra­molecular *i*,*i* + 3 and *i*,*i*-1 C=O⋯H—N hydrogen bonds. The oligomers are linked into chains by N—H⋯O=C hydrogen bonds with the chloro­form solvent mol­ecules inter­calated between the folded chains *via* C—H⋯O=C inter­actions.

## Chemical context   

The title compound is an α,β-hybrid tetra­peptide with alternating proteogenic α-amino acid and ABOC residues. (*S*)-1-amino­bicyclo­[2.2.2]octane-2-carb­oxy­lic acid [(*S*)-ABOC] is a β^2,3,3^-tris­ubstituted bicyclic amino acid which exhibits a high propensity to induce both a reverse turn into short peptides and helices in oligoureas and in α,β-hybrid peptides (Songis *et al.*, 2007 ▸; André *et al.*, 2012 ▸, 2013 ▸; Legrand *et al.*, 2012 ▸, 2014 ▸). In our last study we showed that short oligomers adopted an 11/9 helix, whereas an 18/16 helix was favored for longer oligomers in solution. NMR studies suggested a rapid inter­conversion between the 11/9 helix and the 18/16 helix for oligomers of inter­mediate length. In the solid state, only the 11/9 helix has been observed whatever the length of the oligomers capped by an *i*PrCO and an OBn group (Legrand *et al.*, 2014 ▸).
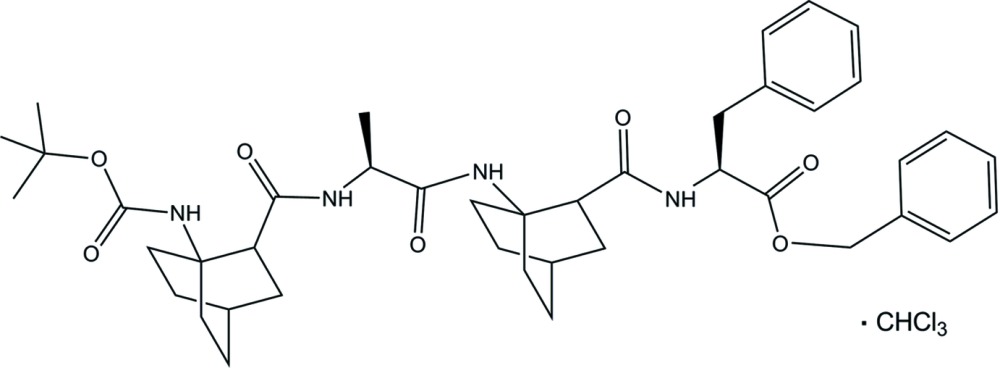


## Structural commentary   

**Figure 1:**
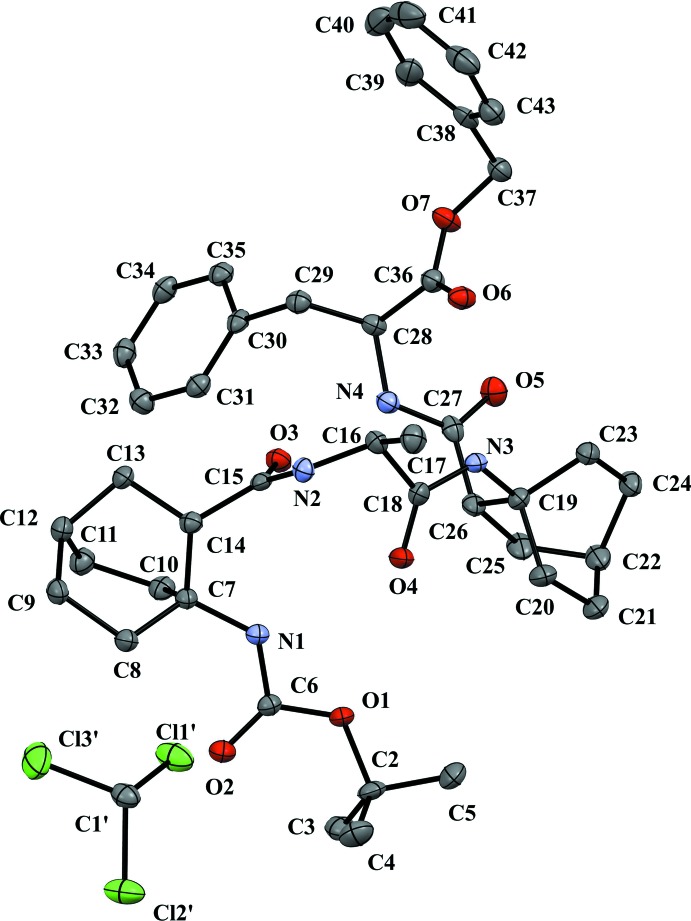
The mol­ecular structure of the title compound showing the atom-numbering scheme. All non-H atoms are represented by 25% probability displacement ellipsoids. H atoms are omitted for clarity.

For the title compound (Fig. 1 ▸), the triclinic unit cell consists of one mol­ecule of α,β-hybrid tetra­mer and one mol­ecule of chloro­form. The oligomer exhibits a right-handed mixed 11/9 helix stabilized by backbone C=O⋯HN hydrogen bonds (Table 1 ▸), forming one C11 pseudocycle between the CO of the β-residue (*i*) and the NH of the α-residue (*i* + 3) and two C9 pseudocycles between the CO of the α-residue (*i*) and the NH of the β-residue (*i* − 1). The backbone torsion angles are quite similar to those of the characteristic 11/9 helix reported in the same α,β-hybrid oligomers (Legrand *et al.*, 2014 ▸) and other α/β-peptides (Lee *et al.*, 2013 ▸).

## Supra­molecular features   

**Figure 2:**
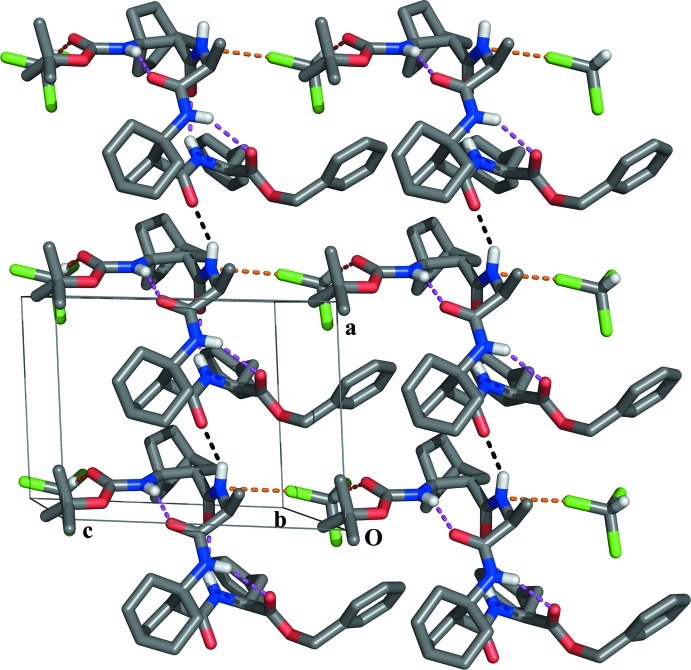
Partial packing view of the title compound in the *ac* plane. Only selected H atoms are shown for clarity. Intra­molecular hydrogen bonds are shown as magenta dashed lines. Inter­molecular strong hydrogen bonds are shown as black dashed lines. Inter­molecular weak hydrogen bonds are shown as red dashed lines. Inter­molecular C—Cl⋯N inter­actions are shown as orange dashed lines.

The inter­molecular inter­action N2—H2⋯O5^i^ (Table 1 ▸) connects the title α,β-hybrid tetra­mer to form infinite chains along the *a*-axis direction (Fig. 2 ▸). In the *ac* plane the chloro­form mol­ecules link the chains *via* a C—Cl⋯N inter­action [Cl⋯N = 3.281 (3) Å] and a C—H⋯O hydrogen bond [C⋯O = 3.071 (4) Å].

## Comparison with related structures   

The crystals of the title compound and those of the same tetra­mer with the N-terminal capping group *i*PrCO instead of Boc are not isomorphous. This latter crystallized in the space group *P*2_1_ with two independent mol­ecules in the asymmetric unit. One independent mol­ecule shows a single fully folded 11/9 helix as the title compound while the hydrogen-bond network is incomplete in the other mol­ecule. The last C9 hydrogen bond between the carbonyl of the Phe residue and the β-residue amide proton was disrupted by the incorporation of a water mol­ecule (Legrand *et al.*, 2014 ▸). This inter­calation of water mol­ecules has already been observed in oligoureas (Legrand *et al.*, 2012 ▸) and highlighted in an enzyme involved in the mitochondrial respiratory chain *i.e.* the mitochondrial bc1 complex. Its bovine crystal structure (Huang *et al.*, 2005 ▸) revealed that an inter­calated water mol­ecule in an α-helix took part in the stabilization of the high potential cytochrome b heme. Usually, α-helices inter­act laterally with their side chains. Water mol­ecules adsorption on an α-helice groove is an alternative tool available to the helical system to inter­act with partners.

For further related articles on hybrid peptides, see: Hayen *et al.* (2004 ▸); Sharma *et al.* (2009 ▸); Vasudev *et al.* (2011 ▸); Berlicki *et al.* (2012 ▸);

## Synthesis and crystallization   

The synthesis of the title compound has recently been reported by Legrand *et al.* (2014 ▸). Single crystals were obtained by slow evaporation of a chloro­form solution.

## Refinement   

Crystal data, data collection and structure refinement details are summarized in Table 2 ▸. All H atoms were located in a difference Fourier map. The C/N-bonded H atoms were placed at calculated positions and refined using a riding model, with C—H = 0.95–1.00 Å and N—H = 0.88 Å. The *U*_iso_(H) parameters were fixed at 1.2*U*_eq_(C, N) for methine, methyl­ene, aromatic groups and NH groups, and at 1.5*U*_eq_(C) for methyl groups.

## Supplementary Material

Crystal structure: contains datablock(s) global, I. DOI: 10.1107/S2056989015016941/is5415sup1.cif

Structure factors: contains datablock(s) I. DOI: 10.1107/S2056989015016941/is5415Isup2.hkl

Supporting information file. DOI: 10.1107/S2056989015016941/is5415Isup3.cml

CCDC reference: 1423394

Additional supporting information:  crystallographic information; 3D view; checkCIF report

## Figures and Tables

**Table 1:** Hydrogen-bond geometry (, )

*D*H*A*	*D*H	H*A*	*D* *A*	*D*H*A*
N1H1O4	0.88	2.16	2.994(4)	157
N2H2O5^i^	0.88	2.12	2.914(3)	150
N3H3*N*O6	0.88	2.51	3.159(3)	131
N4H4O3	0.88	2.20	3.009(3)	153
C1H1O2	1.00	2.09	3.071(4)	167

Symmetry code: (i) 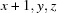.

**Table 2:** Experimental details

Crystal data	
Chemical formula	C_42_H_56_N_4_O_7_CHCl_3_
*M*_r_	848.27
Crystal system, space group	Triclinic, *P*1
Temperature (K)	100
*a*, *b*, *c* ()	9.2194(6), 10.8908(6), 11.8698(7)
, , ()	63.489(2), 86.467(2), 89.069(2)
*V* (^3^)	1064.38(11)
*Z*	1
Radiation type	Mo *K*
(mm^1^)	0.27
Crystal size (mm)	0.4 0.1 0.1

Data collection	
Diffractometer	D8 Venture Bruker
Absorption correction	Multi-scan (*SADABS*; Bruker, 2014 ▸)
*T*_min_, *T*_max_	0.908, 0.963
No. of measured, independent and observed [*I* > 2(*I*)] reflections	42849, 8712, 8015
*R*_int_	0.037
(sin /)_max_ (^1^)	0.626

Refinement	
*R*[*F* ^2^ > 2(*F* ^2^)], *wR*(*F* ^2^), *S*	0.038, 0.087, 1.08
No. of reflections	8712
No. of parameters	518
No. of restraints	3
H-atom treatment	H-atom parameters constrained
_max_, _min_ (e ^3^)	0.30, 0.32
Absolute structure	Flack *x* determined using 3702 quotients [(*I* ^+^)(*I* )]/[(*I* ^+^)+(*I* )] (Parsons *et al.*, 2013 ▸)
Absolute structure parameter	0.006(18)

Computer programs: *APEX2* and *SAINT* (Bruker, 2014 ▸), *SIR2008* (Burla *et al.*, 2007 ▸), *SHELXL2014* (Sheldrick, 2015 ▸), *Mercury* (Macrae *et al.*, 2008 ▸), *pyMOL* (DeLano, 2002 ▸), *WinGX* (Farrugia, 2012 ▸) and *enCIFer* (Allen *et al.*, 2004 ▸).

## supplementary crystallographic information

### Crystal data

**Table:**

C_42_H_56_N_4_O_7_·CHCl_3_	*Z* = 1
*M**_r_* = 848.27	*F*(000) = 450
Triclinic, *P*1	*D*_x_ = 1.323 Mg m^−^^3^
Hall symbol: P 1	Mo *K*α radiation, λ = 0.71073 Å
*a* = 9.2194 (6) Å	Cell parameters from 9972 reflections
*b* = 10.8908 (6) Å	θ = 5.7–52.9°
*c* = 11.8698 (7) Å	µ = 0.27 mm^−^^1^
α = 63.489 (2)°	*T* = 100 K
β = 86.467 (2)°	Needle, colourless
γ = 89.069 (2)°	0.4 × 0.1 × 0.1 mm
*V* = 1064.38 (11) Å^3^	

### Data collection

**Table:**

D8 Venture Bruker diffractometer	8015 reflections with *I* > 2σ(*I*)
φ and ω scans	*R*_int_ = 0.037
Absorption correction: multi-scan (*SADABS*; Bruker, 2014)	θ_max_ = 26.4°, θ_min_ = 2.8°
*T*_min_ = 0.908, *T*_max_ = 0.963	*h* = −11→11
42849 measured reflections	*k* = −13→13
8712 independent reflections	*l* = −14→14

### Refinement

**Table:**

Refinement on *F*^2^	Hydrogen site location: inferred from neighbouring sites
Least-squares matrix: full	H-atom parameters constrained
*R*[*F*^2^ > 2σ(*F*^2^)] = 0.038	*w* = 1/[σ^2^(*F*_o_^2^) + (0.0306*P*)^2^ + 0.6225*P*] where *P* = (*F*_o_^2^ + 2*F*_c_^2^)/3
*wR*(*F*^2^) = 0.087	(Δ/σ)_max_ < 0.001
*S* = 1.08	Δρ_max_ = 0.30 e Å^−^^3^
8712 reflections	Δρ_min_ = −0.32 e Å^−^^3^
518 parameters	Absolute structure: Flack *x* determined using 3702 quotients [(*I*^+^)-(*I*^-^)]/[(*I*^+^)+(*I*^-^)] (Parsons *et al.*, 2013)
3 restraints	Absolute structure parameter: 0.006 (18)

### Special details

**Table:**

Geometry. All e.s.d.'s (except the e.s.d. in the dihedral angle between two l.s. planes) are estimated using the full covariance matrix. The cell e.s.d.'s are taken into account individually in the estimation of e.s.d.'s in distances, angles and torsion angles; correlations between e.s.d.'s in cell parameters are only used when they are defined by crystal symmetry. An approximate (isotropic) treatment of cell e.s.d.'s is used for estimating e.s.d.'s involving l.s. planes.

### Fractional atomic coordinates and isotropic or equivalent isotropic displacement parameters (Å^2^)

**Table:**

	*x*	*y*	*z*	*U*_iso_*/*U*_eq_	
O1	1.0678 (2)	0.1293 (2)	0.8411 (2)	0.0231 (5)	
O2	1.1876 (3)	0.3080 (2)	0.8461 (2)	0.0250 (5)	
O3	0.9584 (2)	0.4011 (2)	0.4043 (2)	0.0217 (5)	
O4	0.9878 (3)	0.1030 (2)	0.5964 (2)	0.0291 (6)	
O6	0.6629 (2)	0.2149 (2)	0.2316 (2)	0.0227 (5)	
O5	0.4408 (2)	0.2042 (2)	0.4540 (2)	0.0255 (5)	
O7	0.4784 (3)	0.3516 (2)	0.1340 (2)	0.0258 (5)	
N1	1.1424 (3)	0.3010 (3)	0.6606 (2)	0.0193 (5)	
H1	1.1204	0.2444	0.6294	0.023*	
N2	1.1419 (3)	0.2721 (3)	0.3845 (2)	0.0184 (5)	
H2	1.2365	0.2643	0.375	0.022*	
N3	0.8189 (3)	0.0590 (3)	0.4865 (2)	0.0182 (5)	
H3N	0.8047	0.0582	0.4143	0.022*	
N4	0.6448 (3)	0.3312 (3)	0.4007 (2)	0.0201 (6)	
H4	0.7247	0.354	0.4241	0.024*	
C2	1.0641 (4)	0.0413 (3)	0.9781 (3)	0.0247 (7)	
C3	0.9992 (4)	0.1148 (4)	1.0520 (3)	0.0320 (8)	
H3A	1.0722	0.1771	1.0557	0.048*	
H3B	0.9688	0.0472	1.1378	0.048*	
H3C	0.9148	0.1676	1.0101	0.048*	
C4	1.2169 (4)	−0.0068 (4)	1.0145 (3)	0.0340 (8)	
H4A	1.2551	−0.0538	0.9655	0.051*	
H4B	1.2152	−0.0701	1.1046	0.051*	
H4C	1.2793	0.0726	0.9968	0.051*	
C5	0.9658 (5)	−0.0760 (4)	0.9937 (3)	0.0337 (8)	
H5A	0.8681	−0.0413	0.9694	0.051*	
H5B	0.9609	−0.1455	1.082	0.051*	
H5C	1.0048	−0.1171	0.9398	0.051*	
C6	1.1374 (3)	0.2516 (3)	0.7879 (3)	0.0188 (6)	
C7	1.1821 (3)	0.4427 (3)	0.5718 (3)	0.0184 (6)	
C8	1.3291 (3)	0.4874 (3)	0.5974 (3)	0.0209 (6)	
H8A	1.4024	0.416	0.6087	0.025*	
H8B	1.3192	0.4987	0.6757	0.025*	
C9	1.3792 (4)	0.6251 (3)	0.4845 (3)	0.0228 (7)	
H9A	1.4202	0.6865	0.5161	0.027*	
H9B	1.4557	0.6079	0.4312	0.027*	
C10	1.0661 (4)	0.5433 (3)	0.5752 (3)	0.0212 (7)	
H10A	1.0424	0.5261	0.6636	0.025*	
H10B	0.9763	0.5293	0.5405	0.025*	
C11	1.1215 (4)	0.6921 (3)	0.4972 (3)	0.0250 (7)	
H11A	1.1538	0.7293	0.554	0.03*	
H11B	1.0423	0.7503	0.4483	0.03*	
C12	1.2495 (4)	0.6932 (3)	0.4068 (3)	0.0229 (7)	
H12	1.2749	0.7893	0.3434	0.027*	
C13	1.2037 (4)	0.6084 (3)	0.3410 (3)	0.0209 (6)	
H13A	1.273	0.6244	0.2685	0.025*	
H13B	1.1059	0.6362	0.3088	0.025*	
C14	1.2014 (3)	0.4545 (3)	0.4365 (3)	0.0180 (6)	
H14	1.2989	0.4167	0.4283	0.022*	
C15	1.0890 (3)	0.3744 (3)	0.4071 (3)	0.0168 (6)	
C16	1.0454 (3)	0.1744 (3)	0.3755 (3)	0.0196 (6)	
H16	0.9842	0.2224	0.3018	0.024*	
C17	1.1352 (4)	0.0646 (3)	0.3577 (3)	0.0259 (7)	
H17A	1.0701	−0.002	0.3515	0.039*	
H17B	1.1951	0.0176	0.4301	0.039*	
H17C	1.1982	0.1078	0.2803	0.039*	
C18	0.9468 (3)	0.1090 (3)	0.4981 (3)	0.0197 (6)	
C19	0.7011 (3)	0.0056 (3)	0.5867 (3)	0.0201 (6)	
C20	0.7585 (4)	−0.0962 (4)	0.7133 (3)	0.0283 (8)	
H20A	0.8161	−0.1678	0.7019	0.034*	
H20B	0.8229	−0.0474	0.7443	0.034*	
C21	0.6307 (4)	−0.1633 (4)	0.8104 (3)	0.0329 (8)	
H21A	0.6228	−0.2616	0.8305	0.04*	
H21B	0.6476	−0.157	0.8892	0.04*	
C22	0.4899 (4)	−0.0907 (4)	0.7570 (3)	0.0291 (8)	
H22	0.4081	−0.1283	0.8229	0.035*	
C23	0.5919 (3)	−0.0714 (3)	0.5489 (3)	0.0215 (7)	
H23A	0.6379	−0.1539	0.5483	0.026*	
H23B	0.5616	−0.0117	0.4629	0.026*	
C24	0.4578 (4)	−0.1144 (4)	0.6431 (3)	0.0251 (7)	
H24A	0.3729	−0.0596	0.6017	0.03*	
H24B	0.4344	−0.2125	0.6711	0.03*	
C25	0.5078 (4)	0.0630 (4)	0.7147 (3)	0.0293 (8)	
H25A	0.5363	0.0786	0.7864	0.035*	
H25B	0.4141	0.1091	0.6871	0.035*	
C26	0.6258 (4)	0.1254 (3)	0.6041 (3)	0.0208 (7)	
H26	0.6998	0.1757	0.6264	0.025*	
C27	0.5618 (3)	0.2238 (3)	0.4817 (3)	0.0199 (7)	
C28	0.5981 (3)	0.4082 (3)	0.2734 (3)	0.0184 (6)	
H28	0.4997	0.4461	0.2785	0.022*	
C29	0.7008 (3)	0.5276 (3)	0.1918 (3)	0.0201 (6)	
H29A	0.8023	0.4964	0.2074	0.024*	
H29B	0.6859	0.5548	0.1019	0.024*	
C30	0.6795 (3)	0.6509 (3)	0.2164 (3)	0.0184 (6)	
C31	0.7622 (3)	0.6728 (3)	0.3003 (3)	0.0215 (7)	
H31	0.8332	0.6077	0.3449	0.026*	
C32	0.7416 (4)	0.7892 (3)	0.3191 (3)	0.0234 (7)	
H32	0.7984	0.8032	0.3766	0.028*	
C33	0.6387 (4)	0.8849 (3)	0.2545 (3)	0.0241 (7)	
H33	0.6258	0.9652	0.2663	0.029*	
C34	0.5550 (4)	0.8624 (3)	0.1729 (3)	0.0232 (7)	
H34	0.4832	0.927	0.1293	0.028*	
C35	0.5748 (4)	0.7466 (3)	0.1541 (3)	0.0215 (7)	
H35	0.5161	0.7323	0.0978	0.026*	
C36	0.5859 (3)	0.3116 (3)	0.2127 (3)	0.0210 (7)	
C37	0.4548 (4)	0.2729 (3)	0.0647 (3)	0.0239 (7)	
H37A	0.3512	0.2783	0.0456	0.029*	
H37B	0.4769	0.1753	0.1182	0.029*	
C38	0.5472 (4)	0.3240 (4)	−0.0556 (3)	0.0245 (7)	
C39	0.5375 (4)	0.4594 (4)	−0.1454 (4)	0.0319 (8)	
H39	0.4758	0.5207	−0.1284	0.038*	
C40	0.6175 (5)	0.5056 (5)	−0.2598 (4)	0.0459 (11)	
H40	0.6111	0.5985	−0.3209	0.055*	
C41	0.7064 (4)	0.4168 (5)	−0.2849 (4)	0.0455 (12)	
H41	0.7596	0.4482	−0.3641	0.055*	
C42	0.7187 (4)	0.2817 (5)	−0.1953 (4)	0.0383 (9)	
H42	0.781	0.2209	−0.2123	0.046*	
C43	0.6393 (4)	0.2362 (4)	−0.0806 (3)	0.0284 (8)	
H43	0.6482	0.144	−0.0186	0.034*	
C1'	1.0834 (4)	0.5291 (4)	0.9249 (3)	0.0258 (7)	
H1'	1.132	0.4645	0.8961	0.031*	
Cl1'	0.89599 (9)	0.52131 (11)	0.91218 (9)	0.0383 (2)	
Cl2'	1.12316 (10)	0.47771 (11)	1.08293 (9)	0.0413 (2)	
Cl3'	1.15106 (13)	0.69484 (11)	0.82768 (10)	0.0495 (3)	

### Atomic displacement parameters (Å^2^)

**Table:**

	*U*^11^	*U*^22^	*U*^33^	*U*^12^	*U*^13^	*U*^23^
O1	0.0315 (13)	0.0226 (12)	0.0123 (11)	−0.0053 (10)	−0.0028 (9)	−0.0049 (9)
O2	0.0315 (13)	0.0266 (12)	0.0178 (11)	−0.0034 (10)	−0.0019 (9)	−0.0107 (10)
O3	0.0192 (12)	0.0247 (12)	0.0221 (12)	0.0005 (9)	−0.0045 (9)	−0.0107 (10)
O4	0.0364 (14)	0.0320 (13)	0.0152 (11)	−0.0141 (11)	−0.0039 (10)	−0.0065 (10)
O6	0.0273 (12)	0.0230 (12)	0.0203 (11)	0.0059 (10)	−0.0049 (9)	−0.0117 (9)
O5	0.0175 (11)	0.0283 (12)	0.0307 (13)	−0.0003 (9)	−0.0002 (9)	−0.0134 (10)
O7	0.0270 (12)	0.0291 (13)	0.0284 (13)	0.0060 (10)	−0.0111 (10)	−0.0183 (11)
N1	0.0228 (14)	0.0205 (13)	0.0144 (13)	−0.0030 (11)	−0.0014 (10)	−0.0076 (11)
N2	0.0145 (12)	0.0208 (13)	0.0193 (13)	−0.0031 (10)	0.0017 (10)	−0.0085 (11)
N3	0.0204 (13)	0.0207 (13)	0.0124 (12)	−0.0024 (11)	−0.0011 (10)	−0.0064 (11)
N4	0.0206 (14)	0.0229 (14)	0.0185 (13)	−0.0018 (11)	−0.0017 (10)	−0.0105 (11)
C2	0.0307 (18)	0.0261 (18)	0.0108 (15)	−0.0006 (14)	−0.0026 (13)	−0.0022 (13)
C3	0.033 (2)	0.043 (2)	0.0204 (17)	−0.0050 (17)	0.0013 (15)	−0.0156 (16)
C4	0.036 (2)	0.034 (2)	0.0256 (19)	0.0067 (16)	−0.0073 (15)	−0.0063 (16)
C5	0.045 (2)	0.0283 (19)	0.0205 (18)	−0.0120 (16)	0.0028 (16)	−0.0042 (15)
C6	0.0206 (15)	0.0198 (15)	0.0149 (14)	−0.0002 (12)	−0.0006 (12)	−0.0069 (12)
C7	0.0208 (16)	0.0194 (15)	0.0153 (15)	−0.0035 (12)	0.0003 (12)	−0.0081 (12)
C8	0.0216 (16)	0.0232 (17)	0.0173 (15)	−0.0031 (13)	−0.0017 (12)	−0.0083 (13)
C9	0.0219 (16)	0.0243 (17)	0.0225 (17)	−0.0079 (13)	0.0009 (13)	−0.0107 (14)
C10	0.0216 (16)	0.0236 (17)	0.0188 (16)	0.0002 (13)	0.0024 (13)	−0.0103 (14)
C11	0.0296 (18)	0.0217 (17)	0.0235 (17)	−0.0009 (14)	0.0018 (14)	−0.0103 (14)
C12	0.0258 (17)	0.0193 (16)	0.0216 (16)	−0.0055 (13)	0.0003 (13)	−0.0075 (13)
C13	0.0227 (16)	0.0205 (16)	0.0159 (15)	−0.0059 (13)	0.0009 (12)	−0.0049 (13)
C14	0.0161 (15)	0.0211 (15)	0.0161 (15)	−0.0010 (12)	0.0002 (12)	−0.0078 (13)
C15	0.0185 (15)	0.0184 (15)	0.0096 (13)	−0.0018 (12)	−0.0005 (11)	−0.0028 (12)
C16	0.0183 (15)	0.0200 (16)	0.0213 (16)	−0.0020 (12)	−0.0027 (12)	−0.0097 (13)
C17	0.0273 (18)	0.0232 (17)	0.0287 (18)	0.0008 (14)	0.0001 (14)	−0.0132 (15)
C18	0.0242 (16)	0.0149 (15)	0.0183 (15)	−0.0009 (12)	−0.0027 (13)	−0.0057 (12)
C19	0.0224 (16)	0.0203 (16)	0.0153 (15)	−0.0048 (12)	0.0004 (12)	−0.0060 (13)
C20	0.036 (2)	0.0286 (18)	0.0153 (16)	−0.0058 (15)	−0.0033 (14)	−0.0054 (14)
C21	0.044 (2)	0.033 (2)	0.0163 (17)	−0.0165 (17)	0.0027 (15)	−0.0052 (15)
C22	0.0334 (19)	0.035 (2)	0.0195 (17)	−0.0143 (16)	0.0082 (14)	−0.0134 (15)
C23	0.0250 (17)	0.0222 (16)	0.0181 (16)	−0.0039 (13)	−0.0003 (13)	−0.0095 (13)
C24	0.0272 (17)	0.0287 (18)	0.0207 (16)	−0.0105 (14)	0.0048 (14)	−0.0126 (14)
C25	0.037 (2)	0.036 (2)	0.0196 (17)	−0.0117 (16)	0.0106 (14)	−0.0177 (15)
C26	0.0248 (16)	0.0233 (16)	0.0167 (15)	−0.0053 (13)	0.0028 (12)	−0.0116 (13)
C27	0.0179 (16)	0.0224 (16)	0.0244 (16)	−0.0025 (13)	0.0035 (13)	−0.0155 (14)
C28	0.0189 (15)	0.0212 (16)	0.0179 (15)	0.0026 (12)	−0.0040 (12)	−0.0110 (13)
C29	0.0196 (16)	0.0223 (16)	0.0186 (15)	0.0004 (13)	0.0003 (12)	−0.0095 (13)
C30	0.0200 (15)	0.0181 (15)	0.0150 (14)	−0.0017 (12)	0.0050 (12)	−0.0060 (12)
C31	0.0211 (16)	0.0214 (16)	0.0193 (16)	0.0001 (13)	0.0015 (12)	−0.0072 (13)
C32	0.0238 (17)	0.0259 (17)	0.0237 (17)	−0.0005 (13)	−0.0011 (13)	−0.0139 (14)
C33	0.0279 (17)	0.0187 (16)	0.0265 (17)	−0.0018 (13)	0.0046 (14)	−0.0116 (14)
C34	0.0229 (16)	0.0182 (16)	0.0238 (17)	0.0028 (13)	0.0011 (13)	−0.0054 (13)
C35	0.0221 (16)	0.0233 (16)	0.0168 (15)	−0.0017 (13)	0.0016 (13)	−0.0073 (13)
C36	0.0208 (16)	0.0229 (17)	0.0197 (16)	−0.0012 (13)	−0.0019 (13)	−0.0097 (13)
C37	0.0280 (18)	0.0236 (17)	0.0233 (17)	−0.0008 (14)	−0.0059 (14)	−0.0126 (14)
C38	0.0238 (17)	0.0284 (17)	0.0238 (17)	−0.0042 (14)	−0.0102 (14)	−0.0128 (15)
C39	0.0273 (19)	0.0280 (19)	0.034 (2)	−0.0013 (15)	−0.0129 (15)	−0.0069 (16)
C40	0.036 (2)	0.045 (2)	0.035 (2)	−0.0161 (19)	−0.0136 (18)	0.0036 (19)
C41	0.027 (2)	0.075 (3)	0.027 (2)	−0.023 (2)	−0.0011 (16)	−0.015 (2)
C42	0.028 (2)	0.061 (3)	0.037 (2)	−0.0103 (18)	−0.0017 (16)	−0.031 (2)
C43	0.0280 (18)	0.0317 (19)	0.0279 (18)	−0.0047 (15)	−0.0034 (14)	−0.0150 (15)
C1'	0.0252 (17)	0.0344 (19)	0.0222 (16)	−0.0014 (14)	0.0004 (13)	−0.0165 (15)
Cl1'	0.0243 (4)	0.0616 (6)	0.0342 (5)	−0.0032 (4)	−0.0015 (4)	−0.0259 (5)
Cl2'	0.0409 (5)	0.0598 (6)	0.0259 (5)	0.0046 (5)	−0.0090 (4)	−0.0208 (5)
Cl3'	0.0607 (7)	0.0457 (6)	0.0387 (6)	−0.0254 (5)	0.0144 (5)	−0.0172 (5)

### Geometric parameters (Å, º)

**Table:**

O1—C6	1.346 (4)	C17—H17B	0.98
O1—C2	1.472 (4)	C17—H17C	0.98
O2—C6	1.222 (4)	C19—C23	1.529 (4)
O3—C15	1.233 (4)	C19—C20	1.536 (4)
O4—C18	1.221 (4)	C19—C26	1.556 (4)
O6—C36	1.203 (4)	C20—C21	1.536 (5)
O5—C27	1.231 (4)	C20—H20A	0.99
O7—C36	1.334 (4)	C20—H20B	0.99
O7—C37	1.454 (4)	C21—C22	1.527 (6)
N1—C6	1.357 (4)	C21—H21A	0.99
N1—C7	1.465 (4)	C21—H21B	0.99
N1—H1	0.88	C22—C25	1.527 (5)
N2—C15	1.336 (4)	C22—C24	1.531 (5)
N2—C16	1.442 (4)	C22—H22	1
N2—H2	0.88	C23—C24	1.542 (4)
N3—C18	1.346 (4)	C23—H23A	0.99
N3—C19	1.475 (4)	C23—H23B	0.99
N3—H3N	0.88	C24—H24A	0.99
N4—C27	1.347 (4)	C24—H24B	0.99
N4—C28	1.452 (4)	C25—C26	1.556 (4)
N4—H4	0.88	C25—H25A	0.99
C2—C5	1.514 (5)	C25—H25B	0.99
C2—C4	1.515 (5)	C26—C27	1.517 (5)
C2—C3	1.521 (5)	C26—H26	1
C3—H3A	0.98	C28—C29	1.526 (4)
C3—H3B	0.98	C28—C36	1.527 (4)
C3—H3C	0.98	C28—H28	1
C4—H4A	0.98	C29—C30	1.503 (4)
C4—H4B	0.98	C29—H29A	0.99
C4—H4C	0.98	C29—H29B	0.99
C5—H5A	0.98	C30—C35	1.391 (5)
C5—H5B	0.98	C30—C31	1.393 (5)
C5—H5C	0.98	C31—C32	1.391 (5)
C7—C10	1.530 (4)	C31—H31	0.95
C7—C8	1.539 (4)	C32—C33	1.385 (5)
C7—C14	1.552 (4)	C32—H32	0.95
C8—C9	1.553 (4)	C33—C34	1.380 (5)
C8—H8A	0.99	C33—H33	0.95
C8—H8B	0.99	C34—C35	1.383 (5)
C9—C12	1.525 (5)	C34—H34	0.95
C9—H9A	0.99	C35—H35	0.95
C9—H9B	0.99	C37—C38	1.494 (5)
C10—C11	1.540 (4)	C37—H37A	0.99
C10—H10A	0.99	C37—H37B	0.99
C10—H10B	0.99	C38—C43	1.385 (5)
C11—C12	1.542 (5)	C38—C39	1.387 (5)
C11—H11A	0.99	C39—C40	1.386 (6)
C11—H11B	0.99	C39—H39	0.95
C12—C13	1.529 (5)	C40—C41	1.378 (7)
C12—H12	1	C40—H40	0.95
C13—C14	1.548 (4)	C41—C42	1.386 (6)
C13—H13A	0.99	C41—H41	0.95
C13—H13B	0.99	C42—C43	1.385 (5)
C14—C15	1.518 (4)	C42—H42	0.95
C14—H14	1	C43—H43	0.95
C16—C17	1.526 (4)	C1'—Cl3'	1.751 (4)
C16—C18	1.545 (4)	C1'—Cl1'	1.751 (3)
C16—H16	1	C1'—Cl2'	1.762 (3)
C17—H17A	0.98	C1'—H1'	1
			
C6—O1—C2	121.4 (2)	C23—C19—C26	110.5 (3)
C36—O7—C37	117.1 (3)	C20—C19—C26	108.6 (3)
C6—N1—C7	124.1 (3)	C21—C20—C19	109.8 (3)
C6—N1—H1	118	C21—C20—H20A	109.7
C7—N1—H1	118	C19—C20—H20A	109.7
C15—N2—C16	120.6 (3)	C21—C20—H20B	109.7
C15—N2—H2	119.7	C19—C20—H20B	109.7
C16—N2—H2	119.7	H20A—C20—H20B	108.2
C18—N3—C19	124.5 (3)	C22—C21—C20	109.6 (3)
C18—N3—H3N	117.8	C22—C21—H21A	109.7
C19—N3—H3N	117.8	C20—C21—H21A	109.7
C27—N4—C28	117.8 (3)	C22—C21—H21B	109.7
C27—N4—H4	121.1	C20—C21—H21B	109.7
C28—N4—H4	121.1	H21A—C21—H21B	108.2
O1—C2—C5	102.2 (3)	C25—C22—C21	109.5 (3)
O1—C2—C4	108.5 (3)	C25—C22—C24	109.2 (3)
C5—C2—C4	111.6 (3)	C21—C22—C24	108.9 (3)
O1—C2—C3	111.8 (3)	C25—C22—H22	109.8
C5—C2—C3	110.5 (3)	C21—C22—H22	109.8
C4—C2—C3	111.7 (3)	C24—C22—H22	109.8
C2—C3—H3A	109.5	C19—C23—C24	109.9 (3)
C2—C3—H3B	109.5	C19—C23—H23A	109.7
H3A—C3—H3B	109.5	C24—C23—H23A	109.7
C2—C3—H3C	109.5	C19—C23—H23B	109.7
H3A—C3—H3C	109.5	C24—C23—H23B	109.7
H3B—C3—H3C	109.5	H23A—C23—H23B	108.2
C2—C4—H4A	109.5	C22—C24—C23	109.3 (3)
C2—C4—H4B	109.5	C22—C24—H24A	109.8
H4A—C4—H4B	109.5	C23—C24—H24A	109.8
C2—C4—H4C	109.5	C22—C24—H24B	109.8
H4A—C4—H4C	109.5	C23—C24—H24B	109.8
H4B—C4—H4C	109.5	H24A—C24—H24B	108.3
C2—C5—H5A	109.5	C22—C25—C26	110.4 (3)
C2—C5—H5B	109.5	C22—C25—H25A	109.6
H5A—C5—H5B	109.5	C26—C25—H25A	109.6
C2—C5—H5C	109.5	C22—C25—H25B	109.6
H5A—C5—H5C	109.5	C26—C25—H25B	109.6
H5B—C5—H5C	109.5	H25A—C25—H25B	108.1
O2—C6—O1	125.0 (3)	C27—C26—C25	112.1 (3)
O2—C6—N1	126.0 (3)	C27—C26—C19	109.6 (2)
O1—C6—N1	109.0 (3)	C25—C26—C19	108.2 (3)
N1—C7—C10	111.5 (2)	C27—C26—H26	109
N1—C7—C8	112.5 (2)	C25—C26—H26	109
C10—C7—C8	108.5 (3)	C19—C26—H26	109
N1—C7—C14	109.2 (2)	O5—C27—N4	120.5 (3)
C10—C7—C14	109.5 (2)	O5—C27—C26	121.8 (3)
C8—C7—C14	105.5 (2)	N4—C27—C26	117.7 (3)
C7—C8—C9	109.4 (3)	N4—C28—C29	112.5 (2)
C7—C8—H8A	109.8	N4—C28—C36	109.2 (2)
C9—C8—H8A	109.8	C29—C28—C36	109.9 (3)
C7—C8—H8B	109.8	N4—C28—H28	108.4
C9—C8—H8B	109.8	C29—C28—H28	108.4
H8A—C8—H8B	108.2	C36—C28—H28	108.4
C12—C9—C8	109.3 (3)	C30—C29—C28	113.4 (3)
C12—C9—H9A	109.8	C30—C29—H29A	108.9
C8—C9—H9A	109.8	C28—C29—H29A	108.9
C12—C9—H9B	109.8	C30—C29—H29B	108.9
C8—C9—H9B	109.8	C28—C29—H29B	108.9
H9A—C9—H9B	108.3	H29A—C29—H29B	107.7
C7—C10—C11	110.1 (3)	C35—C30—C31	118.5 (3)
C7—C10—H10A	109.6	C35—C30—C29	119.6 (3)
C11—C10—H10A	109.6	C31—C30—C29	121.9 (3)
C7—C10—H10B	109.6	C32—C31—C30	120.4 (3)
C11—C10—H10B	109.6	C32—C31—H31	119.8
H10A—C10—H10B	108.2	C30—C31—H31	119.8
C10—C11—C12	108.8 (3)	C33—C32—C31	120.4 (3)
C10—C11—H11A	109.9	C33—C32—H32	119.8
C12—C11—H11A	109.9	C31—C32—H32	119.8
C10—C11—H11B	109.9	C34—C33—C32	119.4 (3)
C12—C11—H11B	109.9	C34—C33—H33	120.3
H11A—C11—H11B	108.3	C32—C33—H33	120.3
C9—C12—C13	108.9 (3)	C33—C34—C35	120.5 (3)
C9—C12—C11	108.5 (3)	C33—C34—H34	119.7
C13—C12—C11	107.8 (3)	C35—C34—H34	119.7
C9—C12—H12	110.5	C34—C35—C30	120.8 (3)
C13—C12—H12	110.5	C34—C35—H35	119.6
C11—C12—H12	110.5	C30—C35—H35	119.6
C12—C13—C14	109.0 (3)	O6—C36—O7	125.0 (3)
C12—C13—H13A	109.9	O6—C36—C28	125.1 (3)
C14—C13—H13A	109.9	O7—C36—C28	109.9 (3)
C12—C13—H13B	109.9	O7—C37—C38	112.1 (3)
C14—C13—H13B	109.9	O7—C37—H37A	109.2
H13A—C13—H13B	108.3	C38—C37—H37A	109.2
C15—C14—C13	111.6 (3)	O7—C37—H37B	109.2
C15—C14—C7	114.1 (2)	C38—C37—H37B	109.2
C13—C14—C7	108.5 (2)	H37A—C37—H37B	107.9
C15—C14—H14	107.4	C43—C38—C39	119.4 (3)
C13—C14—H14	107.4	C43—C38—C37	120.7 (3)
C7—C14—H14	107.4	C39—C38—C37	119.9 (3)
O3—C15—N2	122.2 (3)	C40—C39—C38	120.1 (4)
O3—C15—C14	122.8 (3)	C40—C39—H39	119.9
N2—C15—C14	115.0 (3)	C38—C39—H39	119.9
N2—C16—C17	109.2 (3)	C41—C40—C39	120.0 (4)
N2—C16—C18	108.5 (2)	C41—C40—H40	120
C17—C16—C18	110.5 (3)	C39—C40—H40	120
N2—C16—H16	109.6	C40—C41—C42	120.3 (4)
C17—C16—H16	109.6	C40—C41—H41	119.8
C18—C16—H16	109.6	C42—C41—H41	119.8
C16—C17—H17A	109.5	C43—C42—C41	119.4 (4)
C16—C17—H17B	109.5	C43—C42—H42	120.3
H17A—C17—H17B	109.5	C41—C42—H42	120.3
C16—C17—H17C	109.5	C42—C43—C38	120.6 (4)
H17A—C17—H17C	109.5	C42—C43—H43	119.7
H17B—C17—H17C	109.5	C38—C43—H43	119.7
O4—C18—N3	124.9 (3)	Cl3'—C1'—Cl1'	110.4 (2)
O4—C18—C16	120.3 (3)	Cl3'—C1'—Cl2'	110.83 (19)
N3—C18—C16	114.8 (3)	Cl1'—C1'—Cl2'	110.70 (19)
N3—C19—C23	108.1 (2)	Cl3'—C1'—H1'	108.3
N3—C19—C20	111.5 (3)	Cl1'—C1'—H1'	108.3
C23—C19—C20	107.7 (3)	Cl2'—C1'—H1'	108.3
N3—C19—C26	110.3 (2)		
			
C2—O1—C6—N1	171.0 (3)	N3—C19—C20—C21	−172.7 (3)
C6—N1—C7—C14	170.3 (3)	C23—C19—C20—C21	−54.2 (4)
N1—C7—C14—C15	40.5 (3)	C26—C19—C20—C21	65.5 (3)
C7—C14—C15—N2	−115.8 (3)	C19—C20—C21—C22	−9.3 (4)
C14—C15—N2—C16	169.9 (3)	C20—C21—C22—C25	−54.1 (4)
C15—N2—C16—C18	−56.0 (4)	C20—C21—C22—C24	65.1 (4)
N2—C16—C18—N3	155.4 (3)	N3—C19—C23—C24	−173.3 (3)
C16—C18—N3—C19	−173.2 (3)	C20—C19—C23—C24	66.0 (3)
C18—N3—C19—C26	72.4 (4)	C26—C19—C23—C24	−52.5 (3)
N3—C19—C26—C27	60.2 (3)	C25—C22—C24—C23	66.2 (4)
C19—C26—C27—N4	−92.4 (3)	C21—C22—C24—C23	−53.3 (4)
C26—C27—N4—C28	167.0 (3)	C19—C23—C24—C22	−10.8 (4)
C27—N4—C28—C36	−58.1 (4)	C21—C22—C25—C26	64.3 (4)
N4—C28—C36—O7	147.0 (3)	C24—C22—C25—C26	−54.8 (4)
C6—O1—C2—C5	175.6 (3)	C22—C25—C26—C27	113.0 (3)
C6—O1—C2—C4	−66.3 (4)	C22—C25—C26—C19	−7.9 (4)
C6—O1—C2—C3	57.3 (4)	C23—C19—C26—C27	−59.3 (3)
C2—O1—C6—O2	−9.0 (5)	C20—C19—C26—C27	−177.3 (3)
C7—N1—C6—O2	−13.3 (5)	N3—C19—C26—C25	−177.4 (3)
C7—N1—C6—O1	166.7 (3)	C23—C19—C26—C25	63.2 (3)
C6—N1—C7—C10	−68.6 (4)	C20—C19—C26—C25	−54.8 (3)
C6—N1—C7—C8	53.5 (4)	C28—N4—C27—O5	−10.1 (4)
N1—C7—C8—C9	168.0 (2)	C25—C26—C27—O5	−35.5 (4)
C10—C7—C8—C9	−68.2 (3)	C19—C26—C27—O5	84.6 (4)
C14—C7—C8—C9	49.1 (3)	C25—C26—C27—N4	147.5 (3)
C7—C8—C9—C12	17.7 (4)	C27—N4—C28—C29	179.6 (3)
N1—C7—C10—C11	171.1 (3)	N4—C28—C29—C30	−78.8 (3)
C8—C7—C10—C11	46.7 (3)	C36—C28—C29—C30	159.3 (3)
C14—C7—C10—C11	−68.0 (3)	C28—C29—C30—C35	−85.7 (4)
C7—C10—C11—C12	18.9 (4)	C28—C29—C30—C31	94.4 (3)
C8—C9—C12—C13	−68.8 (3)	C35—C30—C31—C32	−1.1 (5)
C8—C9—C12—C11	48.2 (3)	C29—C30—C31—C32	178.8 (3)
C10—C11—C12—C9	−69.9 (3)	C30—C31—C32—C33	−0.1 (5)
C10—C11—C12—C13	47.9 (4)	C31—C32—C33—C34	1.1 (5)
C9—C12—C13—C14	44.8 (3)	C32—C33—C34—C35	−0.9 (5)
C11—C12—C13—C14	−72.7 (3)	C33—C34—C35—C30	−0.3 (5)
C12—C13—C14—C15	150.0 (3)	C31—C30—C35—C34	1.3 (5)
C12—C13—C14—C7	23.4 (3)	C29—C30—C35—C34	−178.6 (3)
C10—C7—C14—C15	−81.8 (3)	C37—O7—C36—O6	−1.0 (5)
C8—C7—C14—C15	161.6 (3)	C37—O7—C36—C28	178.1 (3)
N1—C7—C14—C13	165.6 (2)	N4—C28—C36—O6	−33.9 (4)
C10—C7—C14—C13	43.3 (3)	C29—C28—C36—O6	90.0 (4)
C8—C7—C14—C13	−73.3 (3)	C29—C28—C36—O7	−89.1 (3)
C16—N2—C15—O3	−10.0 (4)	C36—O7—C37—C38	−87.1 (3)
C13—C14—C15—O3	−59.4 (4)	O7—C37—C38—C43	124.5 (3)
C7—C14—C15—O3	64.1 (4)	O7—C37—C38—C39	−57.7 (4)
C13—C14—C15—N2	120.7 (3)	C43—C38—C39—C40	1.1 (5)
C15—N2—C16—C17	−176.4 (3)	C37—C38—C39—C40	−176.7 (3)
C19—N3—C18—O4	8.4 (5)	C38—C39—C40—C41	0.3 (6)
N2—C16—C18—O4	−26.1 (4)	C39—C40—C41—C42	−1.3 (6)
C17—C16—C18—O4	93.5 (4)	C40—C41—C42—C43	0.8 (6)
C17—C16—C18—N3	−85.0 (3)	C41—C42—C43—C38	0.6 (5)
C18—N3—C19—C23	−166.8 (3)	C39—C38—C43—C42	−1.6 (5)
C18—N3—C19—C20	−48.5 (4)	C37—C38—C43—C42	176.2 (3)

### Hydrogen-bond geometry (Å, º)

**Table:**

*D*—H···*A*	*D*—H	H···*A*	*D*···*A*	*D*—H···*A*
N1—H1···O4	0.88	2.16	2.994 (4)	157
N2—H2···O5^i^	0.88	2.12	2.914 (3)	150
N3—H3*N*···O6	0.88	2.51	3.159 (3)	131
N4—H4···O3	0.88	2.20	3.009 (3)	153
C1′—H1′···O2	1.00	2.09	3.071 (4)	167

Symmetry code: (i) *x*+1, *y*, *z*.
